# Isolation of Viable Single Cells With High Yield and Purity Using a Small Amount of Human Kidney Tissue Biopsy

**DOI:** 10.3389/fcell.2022.822275

**Published:** 2022-05-10

**Authors:** Hasnaa Yaigoub, Hasna Tirichen, Xiaohong Xin, Shuhong Shi, Changxin Wu, Rongshan Li, Yafeng Li

**Affiliations:** ^1^ Institutes of Biomedical Sciences, Shanxi University, Taiyuan, China; ^2^ Department of Nephrology, The Fifth Hospital (Shanxi Provincial People’s Hospital) of Shanxi Medical University, Taiyuan, China; ^3^ Shanxi Provincial Key Laboratory of Kidney Disease, Taiyuan, China; ^4^ Academy of Microbial Ecology, Shanxi Medical University, Taiyuan, China; ^5^ Core Laboratory, The Fifth Hospital (Shanxi Provincial People’s Hospital) of Shanxi Medical University, Taiyuan, China

**Keywords:** human kidney, core needle biopsy, single-cell suspension, tissue dissociation, enzymatic digestion, cell purification

## Abstract

**Objective:** Establishment of an efficient method of preparing human kidney single cell suspension, using a very small amount of tissue puncture.

**Methods:** Samples of human kidney tissue puncture were cut into pieces, and then 80 μL of the digestive enzyme were added to each punctured tissue to induce enzymatic digestion. The enzyme combination is composed of collagenases, DNase and hyaluronidase and the sample was incubated 20 min at 37°C. The obtained cell suspension was filtered through a 70 μm cell strainer, centrifuged at 300 g for 5 min and the supernatant was removed, then the pellet was resuspended in 3 ml of DMEM (Dulbecco’s Modified Eagle’s Medium). Cell suspension was sorted and purified by flow sorting to remove dead cells and obtain a cell suspension with higher viability rate.

**Results:** We found that 1) diverse single cells of human kidney can be obtained by the digestive enzyme, as observed under the light microscope, with different sizes, normal cell morphology and good dispersion. 2) (2-3) × 10^6^ single cells can be extracted from one fresh punctured kidney tissue of about 10 mg, with a cell viability rate of more than 80%.

**Conclusion:** In this work we generated a comprehensive and high-resolution single-cell method, which is simple and efficient for preparing single cell suspension from a minimal amount of human kidney tissue. This method can facilitate the study of renal cell biology and the pathogenesis of kidney diseases.

## Graphical Abstract

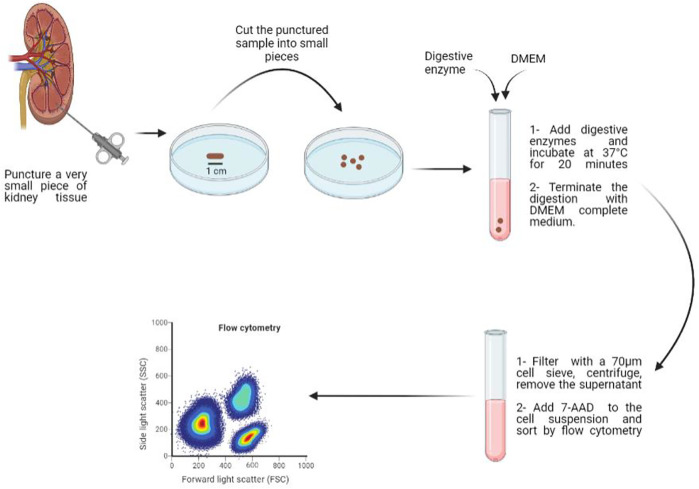

## Introduction

The human kidney is a complex organ that performs multiple functions that are essential for the homeostasis of the human body (Park et al., 2018; Sivakamasundari et al., 2017). It consists of several functionally and anatomically discrete segments, composed of specialized cell types (Park et al., 2018; Schaub et al., 2020). It was reported that some kidney diseases could be cell type-specific, but not restricted to a single type (Schaub et al., 2020; Liao et al., 2020). Therefore, emerging technologies, such as single-cell RNA sequencing, can provide deep understanding of the normal kidney function and the origin of different kidney pathologies at the cellular level. The application of single-cell transcriptomics in kidney diseases has become more widespread as there are continuous advancements in the development of the single-cell RNA sequencing approach and the flow cytometry technique. These techniques can be used for different purposes such as sorting cells with specific functions, identifying disease types, discovering new heterogeneous cell subpopulations, finding genes with specific functions, exploring new therapeutic targets for diseases and evaluating the effects of drug treatments (Stoeckius et al., 2017; Leelatian et al., 2017).

The preparation of single cells is a key part of experiments such as single-cell transcriptomics, flow cytometry, and primary cell culture. However, the degree of differentiation of renal tissue makes the preparation of single cell suspensions difficult (Park et al., 2018). In addition, the limited amount of kidney tissue that could be punctured is challenging for clinicians to conduct further studies. Therefore, establishing an appropriate and efficient method to digest kidney tissue and maximize samples collection from the size-limited kidney punctures is a necessity. In this article, we established an efficient method of preparing a single cell suspension from a minimal amount of human kidney punctures, by exploring enzyme concentration, enzyme action time and cell purification method.

## Materiel and Equipment

### Biological Samples

Human kidney tissue (Shanxi Provincial People’s Hospital).

### Chemicals and Enzyme

• DMEM basal medium (Hyclone, SH30022.01)
• 1×PBS (BOSTER, PYG0021)
• Fetal bovine serum (CELLMA, SA112.02)
• Penicillin and Streptomycin (Gibco, 15140-122)
• Trypan Blue (Gibco, 15250061)
• ACK lysing buffer (Gibco, A1049201)
• AQIX RS-I “Ready to use” Solution (Life Science Group, A1049201)
• FCMase Tissue Digestive Enzyme (HyperCyte, HC0146)
• Collagenase I (Gibco, 17018029)
• Collagenase II (Gibco, 17101015)
• Collagenase IV (Gibco, 17104019)
• Hyaluronidase (Sigma, V900833)
• DNaseI (Sigma, 9003-98-9)
• PI (Yeasen, 40710ES03)
• HypoThermosol FRS Preservation Solution (BioLife Solutions, 07935)
• CS10 cryopreservation solution (BioLife Solutions, 07930)

### Commercial Assays

• MagLive Dead Cell Removal Kit (QDSphere, DCRS-A)

### Equipment

• Inverted phase contrast microscopy for cell culture (LEICA, DM IL LED)
• 70 μm cell strainers sized to fit 50 ml conical tubes (BD, 352340)
• Incubator set at 37°C, 5% CO2 (Heal Force, HF151/212)
• 5 ml conical tubes (BD, 603111)
• 50 ml conical tubes (BD, 352070)
• 100 mm petri dish (NEST, 704201)
• FACS Sorter (BD FACSAria Cell Sorter, FACSCanto0
• EasySepTM (STEMCELL, 18000)
• Sterile surgical instruments and preparation tools (Qingdao Shurtz Biotech Company, Laboratory Anatomical kit)

### Software

• GraphPad Prism 6 for statistical analysis and graphing
• IBM SPSS Statistics 19.0

## Methods

### Kidney Tissue Procurement and Sample Preparation

Fresh human kidney samples were collected at Shanxi Provincial People’s Hospital (Taiyuan, China) from patients with renal cell carcinoma. Samples were transported from the operation room to the laboratory in a sterile bag on ice. In a petri dish, the kidney tissue was cleared of adhering fat and outer membrane, and the fresh sample was stored in tissue preservation solution (AQIX). A section of the tissue block was incubated in HypoThermosol FRS preservation solution for 30 min, supplemented with 1 ml of CS10 cryopreservation solution, and stored at −80°C overnight, then transferred to liquid nitrogen. Another section of the tissue block was placed in a 50 ml centrifuge tube, cut into small pieces with a puncture needle, and weighed separately. The obtained samples were placed in a 5 ml centrifuge tube, washed with 3 ml of PBS, centrifuged at 300 g for 5 min, and then the supernatant was removed. The punctured sample was cut with surgical scissors and washed again with PBS. After discarding the supernatant, the digestive enzyme was used to further digest the sticky clumps of cells for 20 min at 37°C and the digestion was stopped using DMEM complete medium. The resulting cell suspension was then filtered through a 70 μm cell strainer and centrifuged at 300 g for 5 min, the supernatant was removed and finally, the pellet was resuspended in DMEM medium.

### FCMase Enzyme Concentration

Different concentrations of FCMase enzyme (6, 7, 8, and 9 μL/mg) were added to the kidney tissue samples and then incubated at 37°C for 20 min. During this incubation, samples were mixed every 5 min.

### Duration of the FCMase Digestion

The FCMase digestive enzyme was added to the kidney tissue samples at a concentration of 8 μL/mg, which means that 80 μL of FCMase enzyme was added to one punctured tissue (10 mg). Subsequently, the tissue samples were digested at 37°C for 10 min, 20 min, and 30 min and were mixed every 5 min.

### Collagenase Types and Concentrations

A stock solution of 8 mg/ml was prepared for collagenase I, collagenase II, and collagenase IV by mixing an amount of 126.6, 36.2, and 42.6 mg, respectively, with 15.83, 4.53, and 5.33 ml of DMEM high glucose medium, respectively. Each collagenase type was added to the kidney puncture samples, at a ratio of 1/8 mg/ml, using the following concentrations 0.125, 0.25, 0.5, 1, 2, and 4 mg/ml, and incubated at 37°C for 20 min while mixing every 5 min.

### Collagenases Combinations

The L_9_ (3^4^) orthogonal table was used to design nine collagenase combination groups. The type, concentration, and formulation of collagenase used are shown in Table 1. DNase I (0.1 mg/ml) and hyaluronidase (0.1 mg/ml) were added to each collagenase combination and incubated at 37°C for 20 min with mixing every 5 min. Cell count and viability were measured. Liberase TM and FCMase were used for comparison (Table 1).

**TABLE 1:** Orthogonal design of different digestive enzyme combinations.

Group	Col Ⅱ (mg/ml)	Col IV (mg/ml)	Col Ⅰ (mg/ml)
A	1	0.125	0.125
B	1	0.25	0.25
C	1	0.5	0.5
D	2	0.125	0.25
E	2	0.25	0.5
F	2	0.5	0.125
G	3	0.125	0.5
H	3	0.25	0.125
I	3	0.5	0.25
J	Liberase TM 0.025 mg/ml + 0.05 mg/ml DNase Ⅰ		
K	FCMase +0.1 mg/ml DNase Ⅰ		

### Cell Purification Methods

After preparing the single cell suspension with fresh human kidney tissue, five experimental groups were used to test the following cell purification methods: magnetic beads cell sorting (group 1), DNA purification magnetic beads (group 2), centrifugation on percoll density gradient (group 3), fluorescence-activated cell sorting (FACS) (group 4), and a control group, in which no purification method was tested. For group 1, 50 μL/ml of cell magnetic beads were added for 5 min at room temperature, incubated on a magnetic stand for 10 min, then washed twice with PBS before resuspension. For group 2, DNA purification magnetic beads were added for 5 min and incubated on a magnetic stand for 10 min at room temperature. The liquid was then collected, centrifuged, and resuspended in PBS. For group 3, 3 ml of 30% percoll working solution were added and 1 ml of the cell suspension was placed on the top layer of the 30% percoll solution for 5 min at room temperature, then centrifuged at 400 g for 40 min. After stratification, the top liquid layer was discarded and cells were washed twice with PBS and resuspended. Finally, for group 4, 1 ml of flow cytometry staining buffer and 10 μL of 7-AAD were added to the cell suspension and incubated for 10 min, then sorted by flow cytometry. The effect of these purification methods on the cell count and cell viability was observed.

### Red Blood Cell Lysate and Cell Viability

After preparing a single cell suspension with fresh human kidney tissue, we used two groups, the control group untreated, and the experimental group to which we added 3 ml of ACK (Ammonium-Chloride-Potassium) Lysing buffer. It was then incubated on ice for 5 min, and centrifuged to remove the supernatant.

### Testing Indicators and Statistical Analysis

Single cell suspension (1 ml) was centrifuged and the cell pellet was resuspended with 1 ml of flow cytometry staining buffer. 2 μL of PI labeled cells were added and the result of cell viability of the obtained single cells was detected using flow cytomety. Prepared single cells were counted under a microscope. Trypan blue 0.4% was added to the cell suspension at a ratio of 1/9 for 3 min, and the death rate of cells was recorded.

Graphpad prism 6 software was used for data statistical analysis and graphing, and SPSS software was used for statistical analysis. All measurement data are expressed as (mean ± SD).

## Results

### Effect of FCMase Enzyme Concentration on the Preparation of the Single Cell Suspension

**FIGURE 1:**
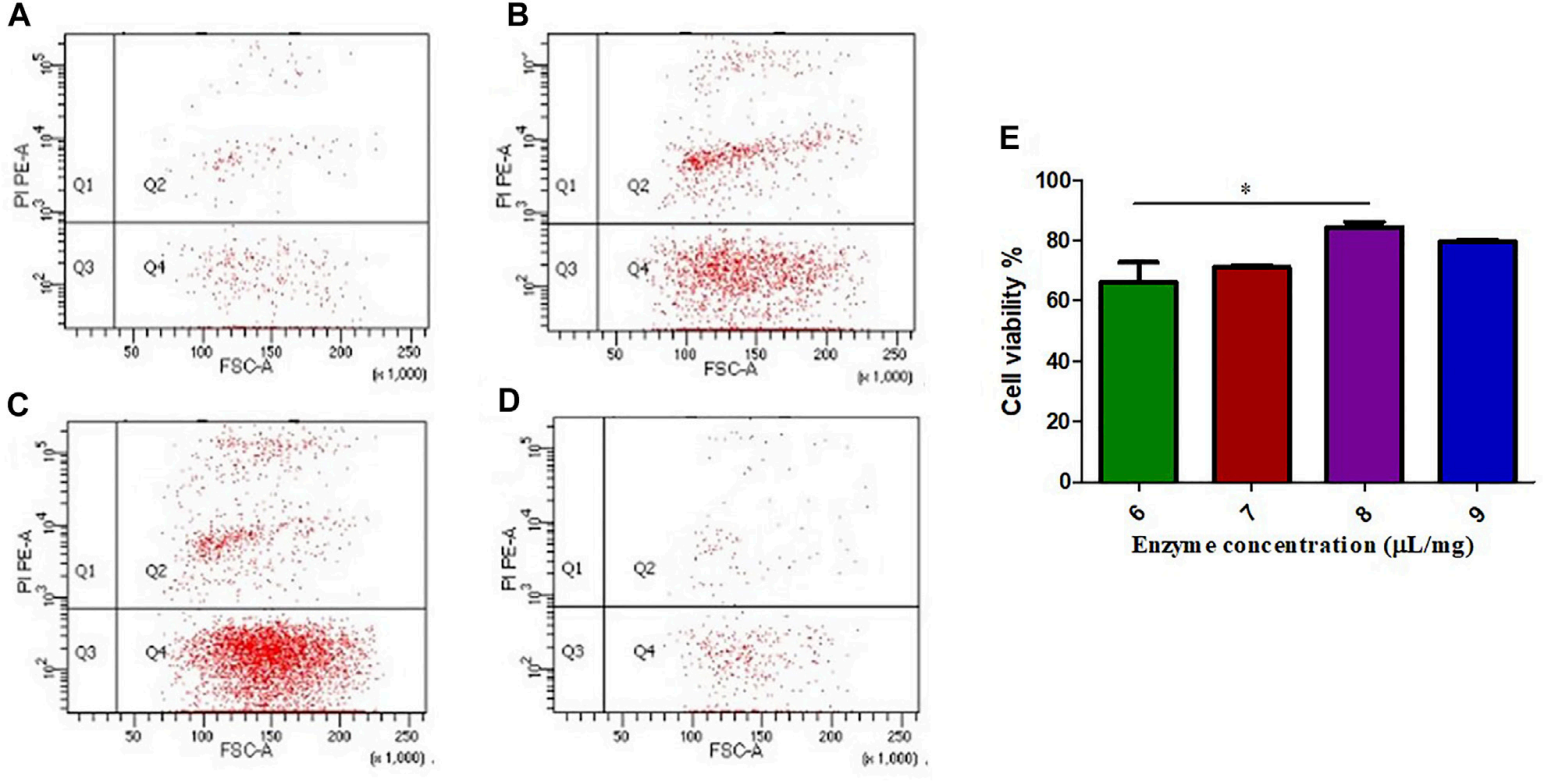
The effect of FCMase enzyme concentrations on cell viability during the preparation of single cell suspension. **(A)** 6 μL/mg, **(B)** 7 μL/mg, **(C)** 8 μL/mg, **(D)** 9 μL/mg, of FCMase enzyme. **(E)** Statistical comparison of the effect of FCMase enzyme concentrations on cell viability. Data are presented as means ± SD; *p value <0.05.

The different FCMase concentrations that have been tested in the present study have different effects on cell viability. The single cell suspension prepared with the concentration 8 μL/mg resulted in the highest cell viability exceeding 80% in comparison with other concentrations (Figure 1). The concentration 6 μL/mg has the lowest cell viability rate (Figure 1).

### Effect of the Duration of the FCMase Digestion on the Preparation of Single Cell Suspension

**FIGURE 2:**
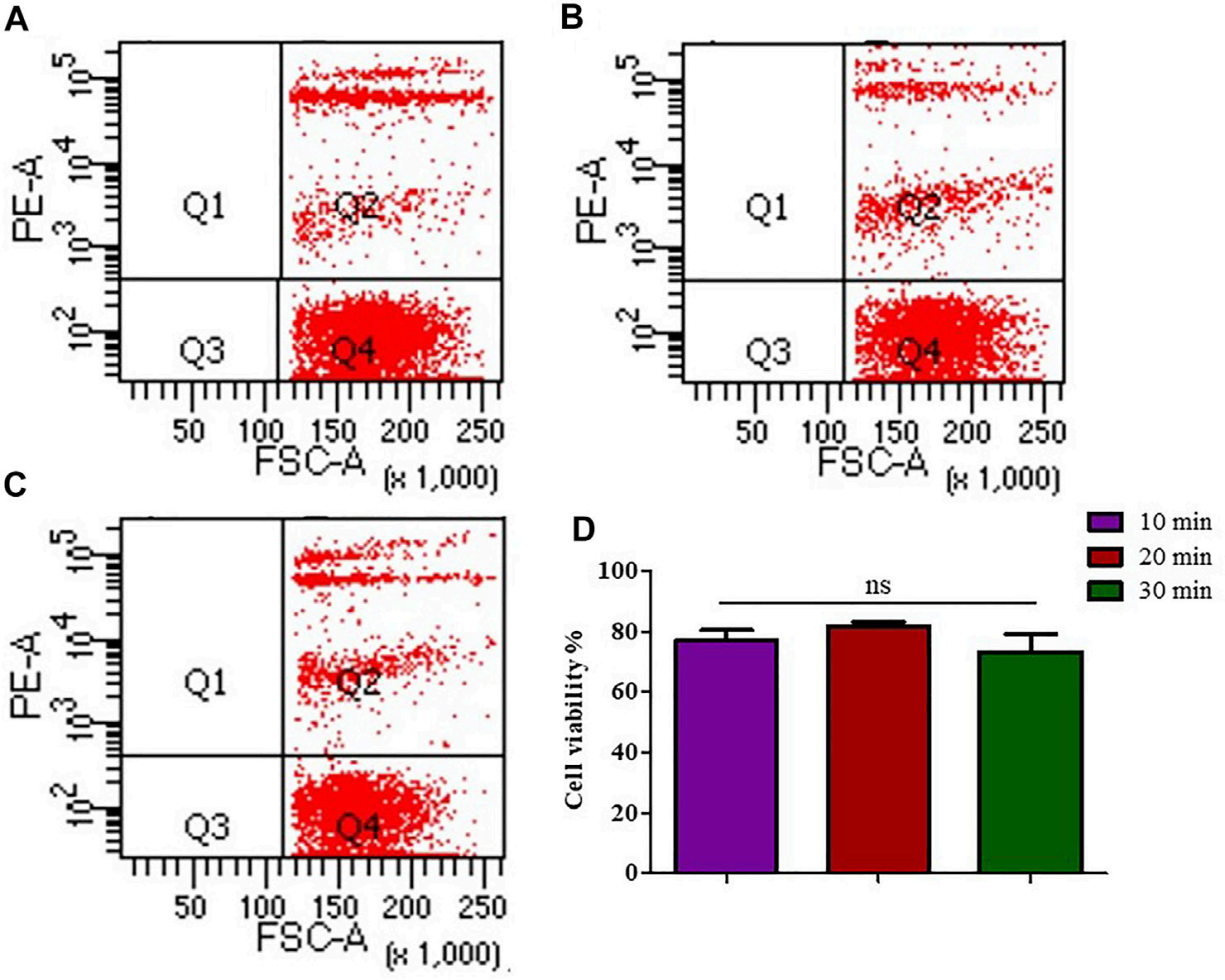
The effect of FCMase enzyme digestion time durations on cell viability during the preparation of single cell suspension. **(A)** 10 min, **(B)** 20 min, **(C)** 30 min, of FCMase enzyme digestion duration. **(D)** Statistical comparison of the effect of FCMase enzyme digestion time durations on cell viability. Data are presented as means ± SD; ns *p* value = 0.1111.

The single cell suspension prepared with the concentration of 8 μL/mg of FCMase showed no significant difference in cell viability in the three incubation times that have been tested. However, the cell viability rate at 20 min of incubation remains the highest (Figure 2).

### Effect of Different Collagenases on the Preparation of Single Cell Suspension

**FIGURE 3:**
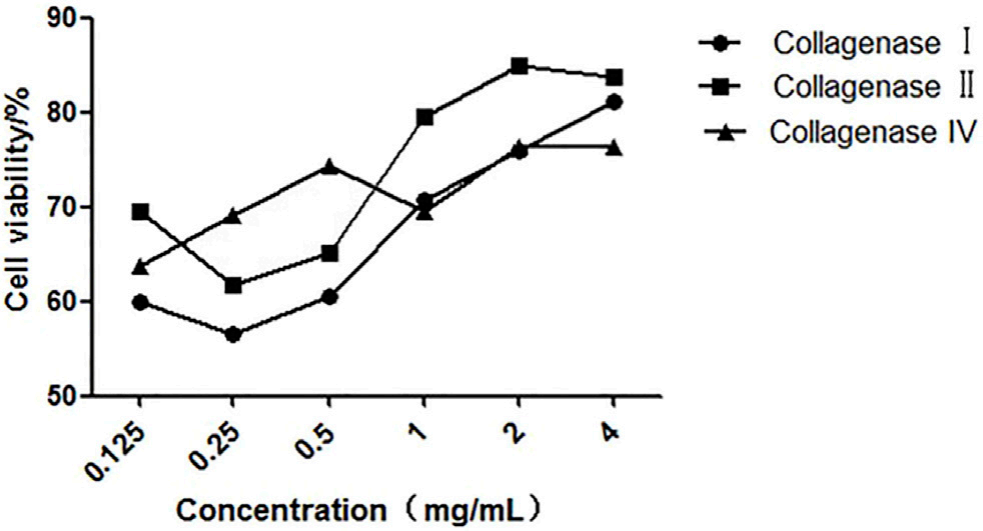
The effect of different concentrations of collagenases on cell viability during kidney tissue digestion.

Different collagenases used to digest kidney tissue showed different effects. As shown in Figure 3, we found that the cell viability is the highest (more than 85%), when we used 2 mg/ml collagenase II on single cells from digested kidney tissue. Also, compared to the other collagenases, collagenase type IV has better effect on kidney tissue dissociation at a low concentration. Furthermore, collagenase II has shown higher digestive activity compared to collagenase I at all tested concentrations. At a concentration greater than 1 mg/ml, we observed that the activity of the three collagenases on the single cells, digested from kidney tissue, is higher than that of FCMase enzyme (data not shown).

### Effect of Collagenase Combinations on the Preparation of Single Cell Suspension

**FIGURE 4:**
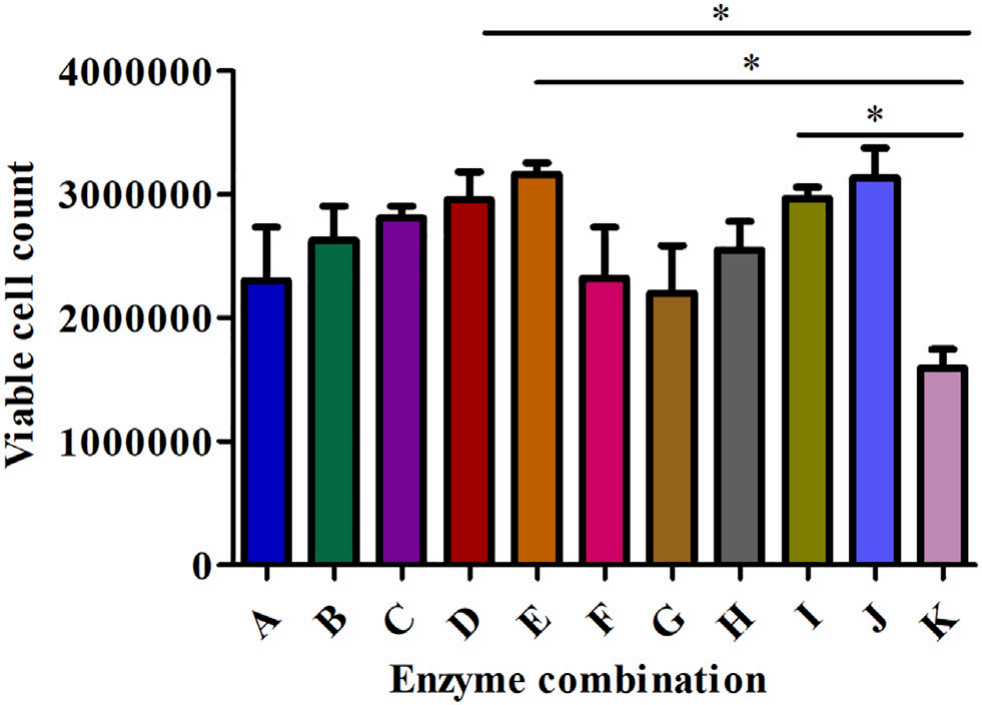
The effect of different enzyme combinations on cell count during kidney tissue digestion. Data are presented as means ± SD; *p value = 0.0129.

**FIGURE 5:**
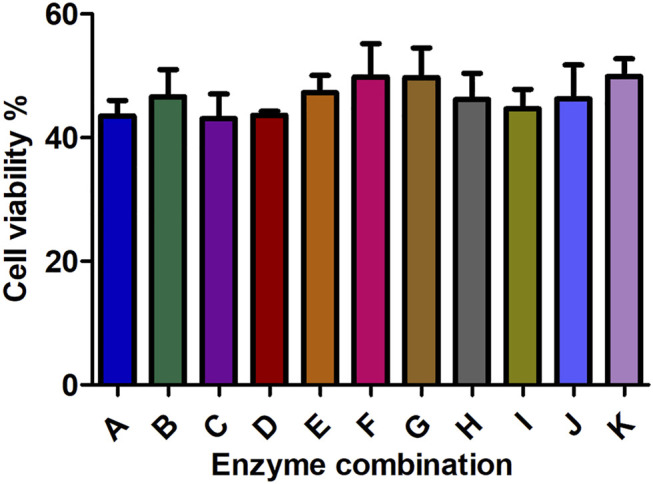
The effect of different enzyme combinations on cell viability during kidney tissue digestion. Data are presented as means ± SD; ns *p* value = 0.2953.

We investigated the effect of different concentrations of the three types of collagenases I, II, and IV on the count and viability of digested kidney cells. Detailed results are shown in Figures 4, 5.

Collagenase I is the main factor that generates the highest cell count (458888.889) in the suspension compared to collagenase II (240000) and collagenase IV (292222.222) as shown in Table 2. Regarding cell viability, collagenase II generated the highest rate of viable cells (2.487) followed by collagenase I and collagenase IV (1.706 and 1.055 respectively) as reported in Table 3.

**TABLE 2:** Range analysis of collagenases combinations on cell count during kidney tissue digestion.

	Col Ⅱ (mg/ml)	Col IV (mg/ml)	Col Ⅰ (mg/ml)
K_1_	2578888.889	2486666.667	2391111.111
K_2_	2812222.222	2778888.889	2850000
K_3_	2572222.222	2697777.778	2722222.222
R	240000	292222.222	458888.889

**TABLE 3:** Range analysis of collagenases combinations on cell viability during kidney tissue digestion.

	Col Ⅱ (mg/ml)	Col IV (mg/ml)	Col Ⅰ (mg/ml)
K_1_	44.409	45.629	46.506
K_2_	46.896	46.684	44.971
K_3_	46.849	45.84	46.677
R	2.487	1.055	1.706

All collagenase combinations that have been used in this study are listed in Table 1 (A-K) and results are presented in Figure 5. The collagenase combination used in group E (2 mg/ml collagenase Ⅱ + 0.25 mg/ml collagenase IV + 0.5 mg/ml collagenase Ⅰ) resulted in the highest cell count in comparison with other collagenase combinations (group A- group I). There is a significant difference in cell count between groups E and K, but not between E and J. However, no significant effect was observed on the cell viability of digested single cells (Figure 5).

### Effect of Different Purification Methods on the Preparation of Single Cell Suspension

**FIGURE 6:**
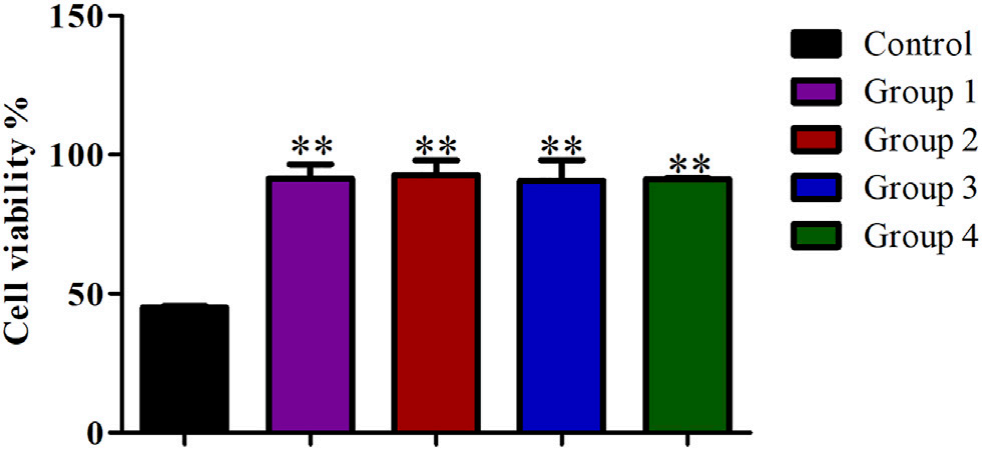
The effect of different cell purification methods on single cell viability. Data are presented as means ± SD; **p value <0.05.

**FIGURE 7:**
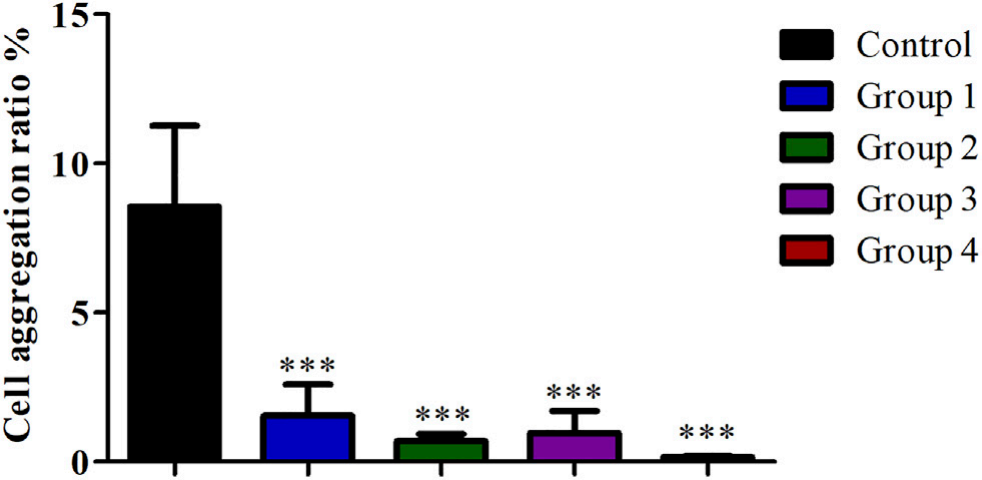
The effect of different cell purification methods on cell aggregation ratio. Data are presented as means ± SD; ***p value <0.05.

**FIGURE 8:**
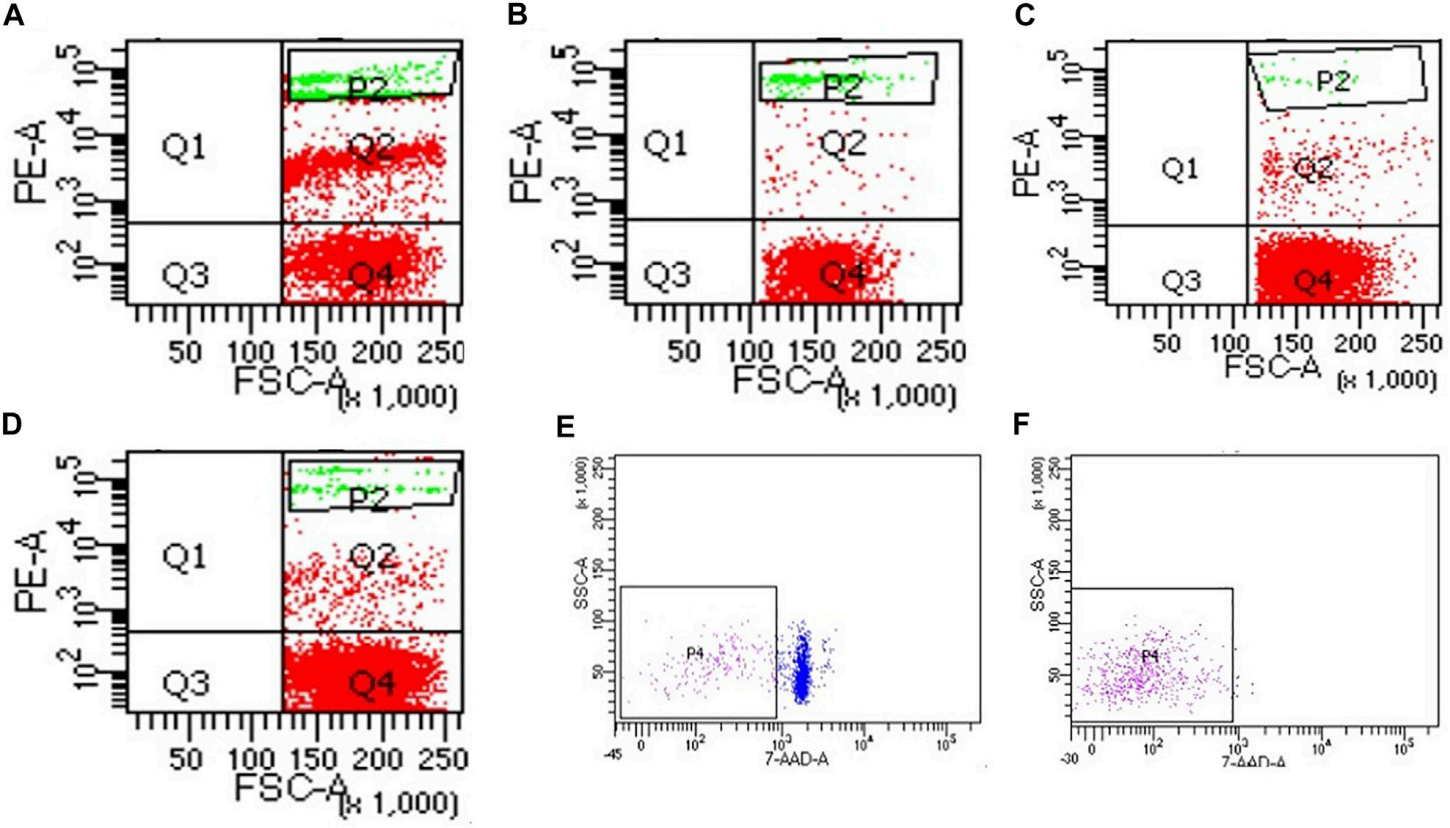
The effect of different cell purification methods on cell aggregation. **(A)** Control, **(B)** Group 1 (magnetic beads cell sorting), **(C)** Group 2 (DNA purification magnetic beads), **(D)** Group 3 (Centrifugation on Percoll density gradient), **(E)** Control of group 4, **(F)** Group 4 (FACS).

Compared with the control group, the cell viability in the four purification groups is significantly increased by more than 30% (Figure 6), and the cell aggregation ratio is significantly decreased (Figure 7). The difference between results obtained by DNA purification magnetic beads and FACS is minimal. Figure 8 reveals that DNA purification magnetic beads can reduce most of the cell aggregations and separate them into single cells.

### Effect of the Red Blood Cell Lysate on the Preparation of Single Cell Suspension

**FIGURE 9:**
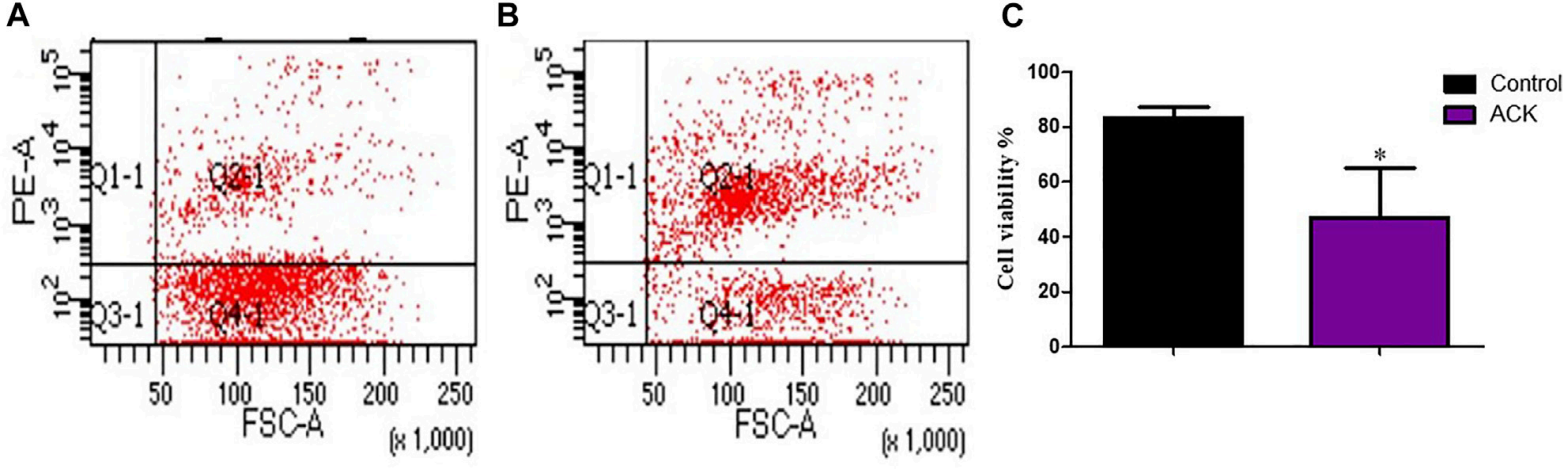
The effect of the red blood cell lysate on cell viability during the preparation of single cell suspension. **(A)** control group, **(B)** group treated with ACK lysing buffer, **(C)** Statistical comparison of the effect of ACK lysing buffer on cell viability. Data are presented as means ± SD; *p value = 0.0266.

The treatment of the samples with ACK lysing buffer resulted in a higher number of dead cells, which indicates that red blood cells were removed from the single cell suspension (Figure 9).

## Discussion

Recently, the prevalence of kidney disease has been increasing worldwide (Lv and Zhang, 2019). Experimental research in this field has gradually shifted from the animal level to the human single cell and molecular level. To detect certain cell type from the complex kidney tissue, it is necessary to dissociate it to obtain human kidney single cells (Denisenko et al., 2020; Reichard and Asosingh, 2019). The preparation of a single cell suspension is the key step in *in vitro* cell culture and in cell biology research (Alberts et al., 2002). This requires a simple, stable, and efficient method to separate kidney cells in order to provide an efficient platform for scientific research.

In the process of preparing a single cell suspension, it is necessary to ensure that the cells remain as intact as possible. The prepared single cell suspension should contain a minimum amount of DNA released by dead cells or fragmented cells (Wilson et al., 2019; Reichard and Asosingh, 2019). In this research method, the kidney tissue was cut into pieces in the EP tube to avoid the loss of sample volume and washed with PBS twice to remove the cellular impurities. Then, the enzymatic digestion was carried out followed by the lysis of red blood cells. Afterwards, density gradient separation was performed, followed by removal of dead cells to obtain single cells using the magnetic beads or the flow cytometry 7-AAD negative sorting.

At present, enzymatic digestion is most commonly used to digest the kidney tissue. Neutral protease can effectively degrade the connexin between cells and extracellular matrix, and has almost no effect on cell-cell connections, so it is often used for the separation of epidermis and dermis (Rao and Zaidel-Bar, 2016). The major extracellular matrix component in dermal tissue is collagen (Weinstein and Boucek, 1960). For this reason, we sought to use a combination of different collagenases to digest the kidney tissue. This combination resulted in an optimal effect on the kidney tissue dissociation which is in line with the findings of a research conducted on degenerated intervertebral discs to isolate nucleus pulposus cells where collagenase I and II were used in combination and their effect was stronger than the use of each type separately (Feng et al., 2018).

During tissue digestion, it is important to maximize the contact surface between the tissues and digestive enzymes (Reichard and Asosingh, 2019). Therefore, we mixed the samples every 5 min during the incubation, thus digesting the maximum of the tissue and obtaining more single cells.

A number of studies in single cell isolation use Liberase TM and DNase Ⅰ enzyme to dissociate the tissue and obtain a single cell suspension (Lauder et al., 2021; Yu et al., 2020). Compared with other digestive enzymes, Liberase TM allows the isolation of highly viable cells, but has resulted in incomplete tissue digestion (Schmidt et al., 2018; Dolmans et al., 2006). There are two main reasons for cell aggregation that are: incomplete digestion of the connections between cells and the cell aggregation induced by free DNA strands released by cell rupture (Reichard and Asosingh, 2019). Therefore, adding DNase I to the digestive enzymes may reduce cell debris and increase the count of single cell in the suspension (Reichard and Asosingh, 2019). The final enzyme we added to digestive combination is the hyaluronidase as it contributes to the digestion of the extracellular matrix in solid tissues by degrading the hyaluronan (Stern and Jedrzejas., 2006).

It is recommended to use ACK lysing buffer if the cell suspension contains red blood cells or platelets (Leelatian et al., 2017). However, when using it, we have found that it causes damage to cells as evidenced by the increase in dead cells and cell debris. Therefore, we suggest not using it for small volume samples.

Another process that could interfere with the quality of the cell suspension is the centrifugation force. A very high centrifugal force increases the cell death rate, and a very low centrifugation rate increases the number of cell debris in the prepared cell suspension (Reichard and Asosingh, 2019).

Furthermore, clinicians have the constraint of a limited number of punctures and the small amount of kidney tissue that could be punctured. A minimum of two to three specimens are needed for light microscopy, electron microscopy, and immunofluorescence analysis. We consider that the novelty of the method we have established is the very small amount (10 mg) of renal puncture to obtain a high rate of cell viability (above 80%) and purity. The number of single cells obtained by separating a small amount of kidney tissue by this method can meet the needs of general renal cell biology research, and it is convenient to apply in experiments such as flow cytometry and cell culture. It is also suitable to use in single-cell transcriptomics analysis for in-depth understanding of the changes in the level of single cells during the kidney disease process. In addition, in this method we used a new combination of digestive enzymes including collagenase I, collagenase II, collagenase IV, DNase I, and hyaluronidase in order to maximize the resulting amount of single cells from the biopsy sample. Other published studies, in human or mice, used either collagenase I, collagenase II, or collagenase I combined with DNase I (Dumas et al., 2021; Zhang et al., 2021; Zimmerman et al., 2019).

## Conclusion

The increase in studies applying single cell analysis requires an efficient and simple single cell isolation method. In this study, we established an efficient method of viable single cell isolation from human kidney tissue puncture based on enzymatic tissue dissociation. A minimal amount of kidney tissue sample (10 mg) was dissociated into a single cell suspension using the following formula: 2 mg/ml collagenase Ⅱ + 0.25 mg/ml collagenase IV + 0.5 mg/ml collagenase Ⅰ + 0.1 mg/ml DNase I + 0.1 mg/ml hyaluronidase, then sorted and purified by flow sorting to remove dead cells and obtain a cell suspension with higher viability rate (greater than 80%).

## Data Availability

The raw data supporting the conclusion of this article will be made available by the authors, without undue reservation.
